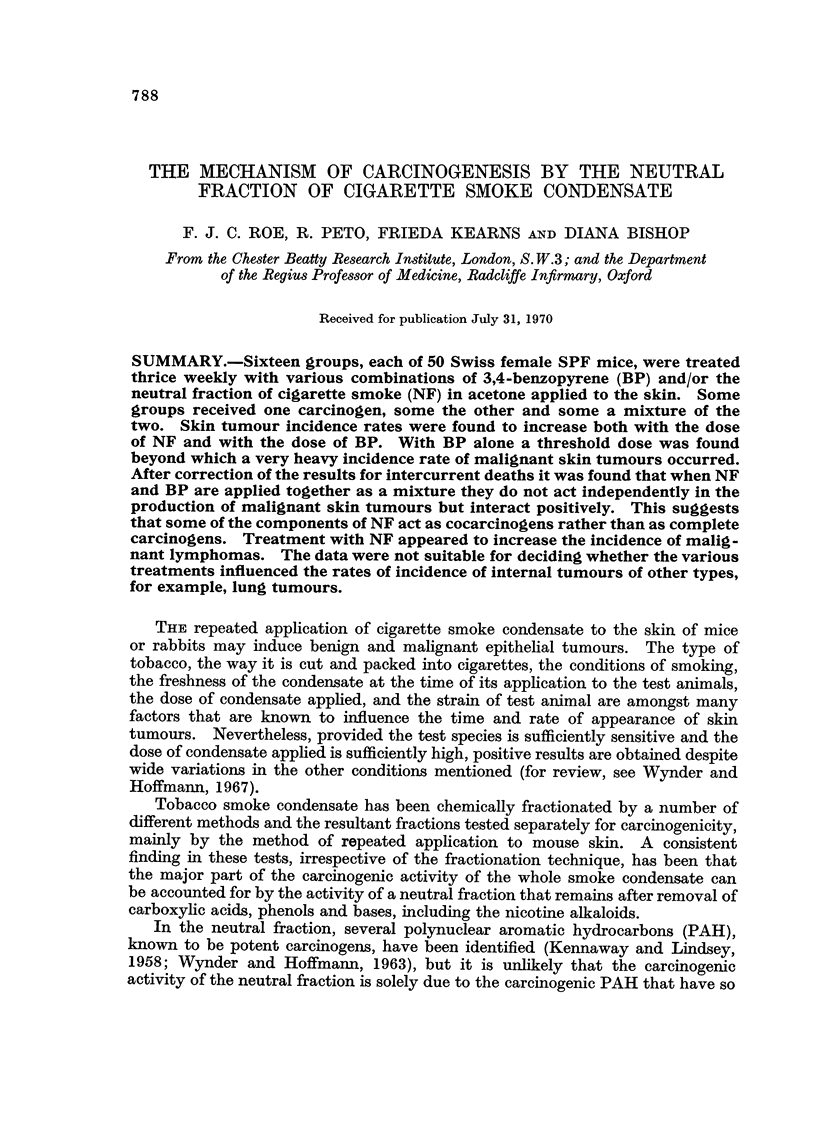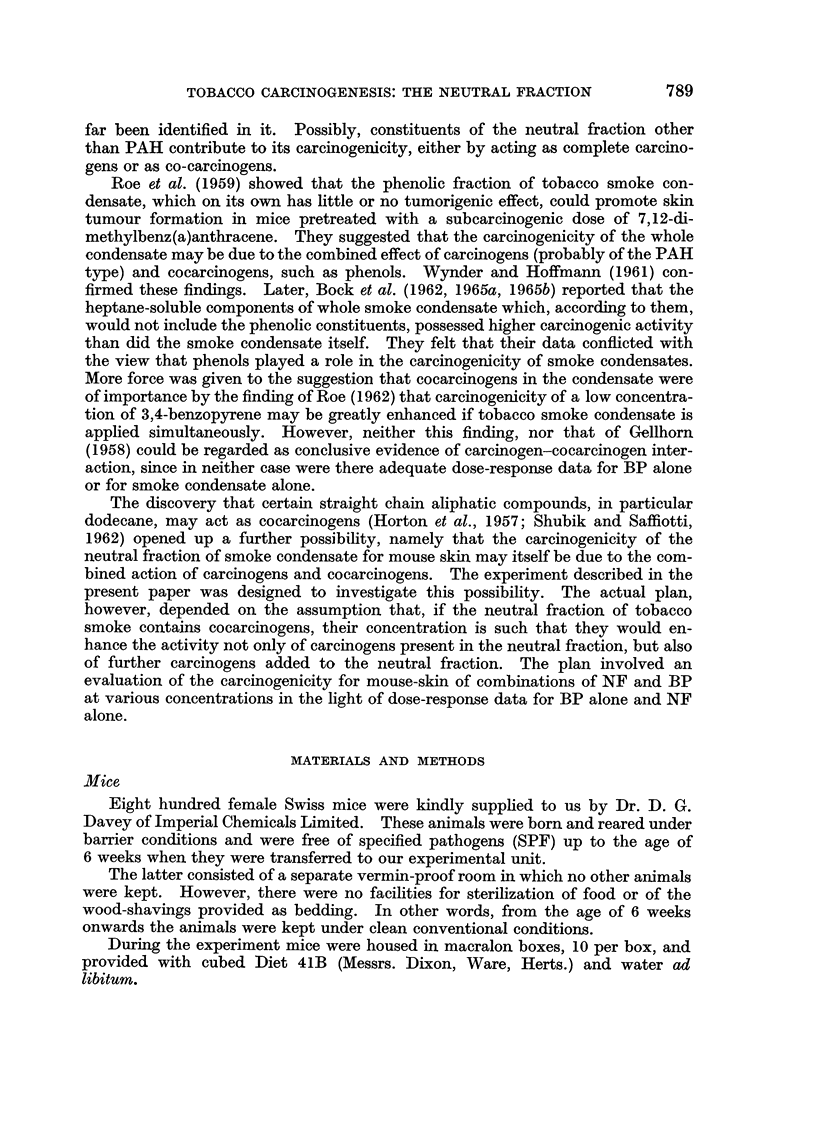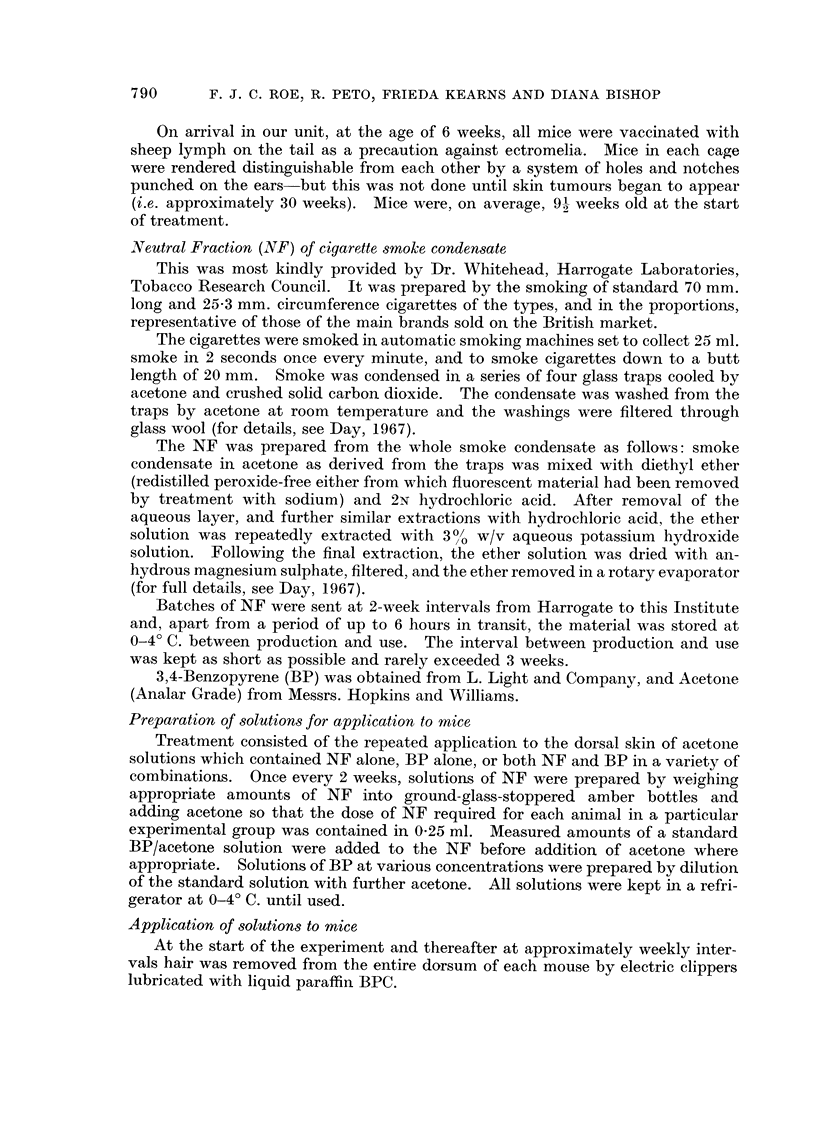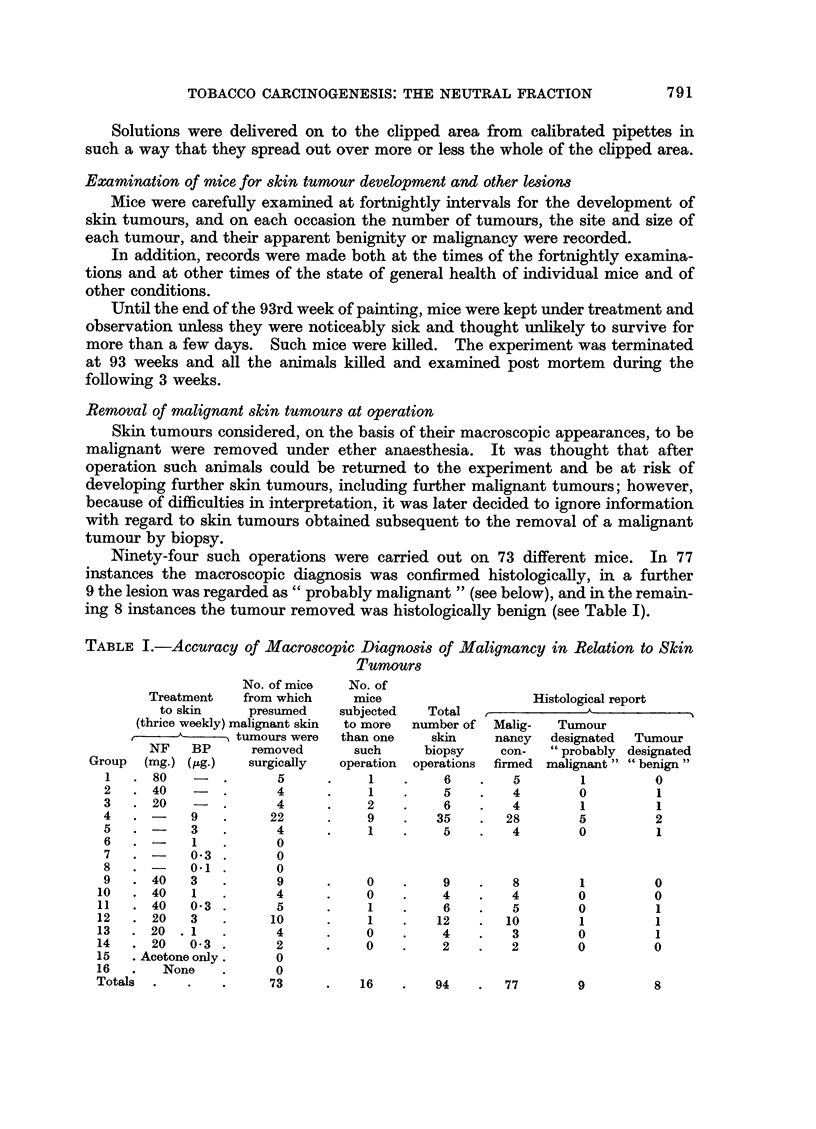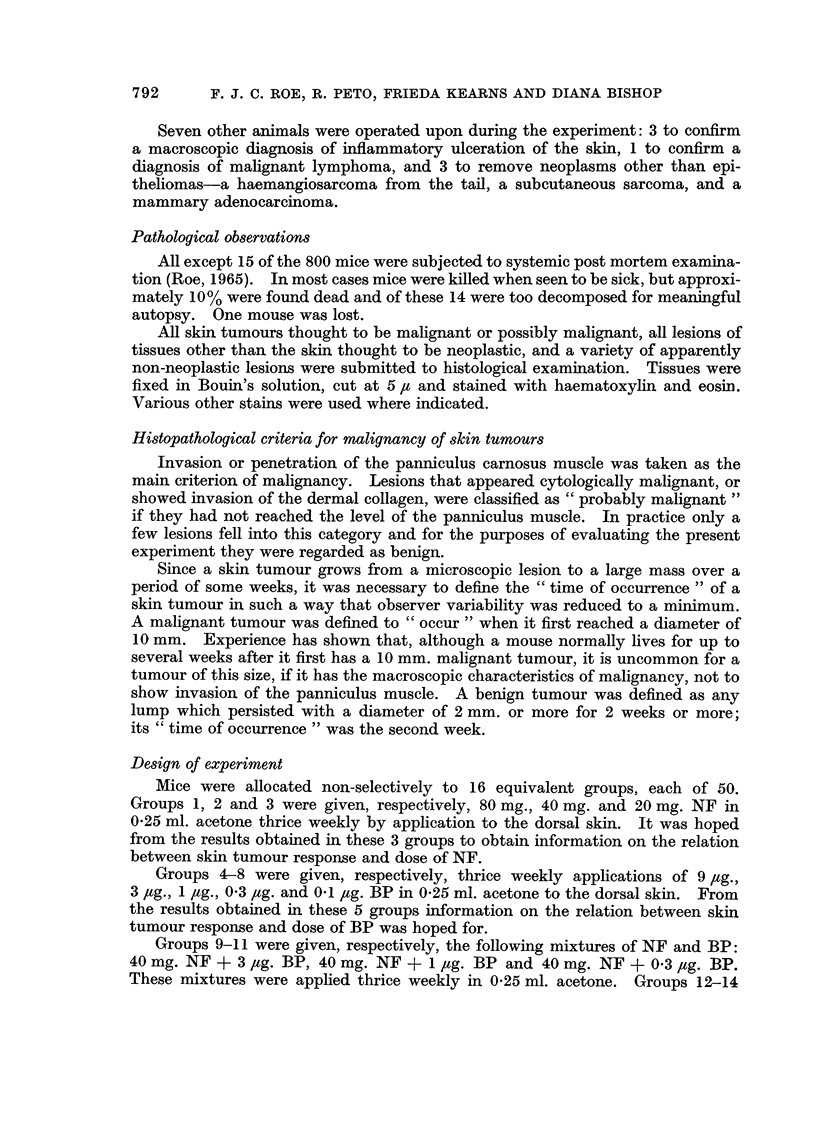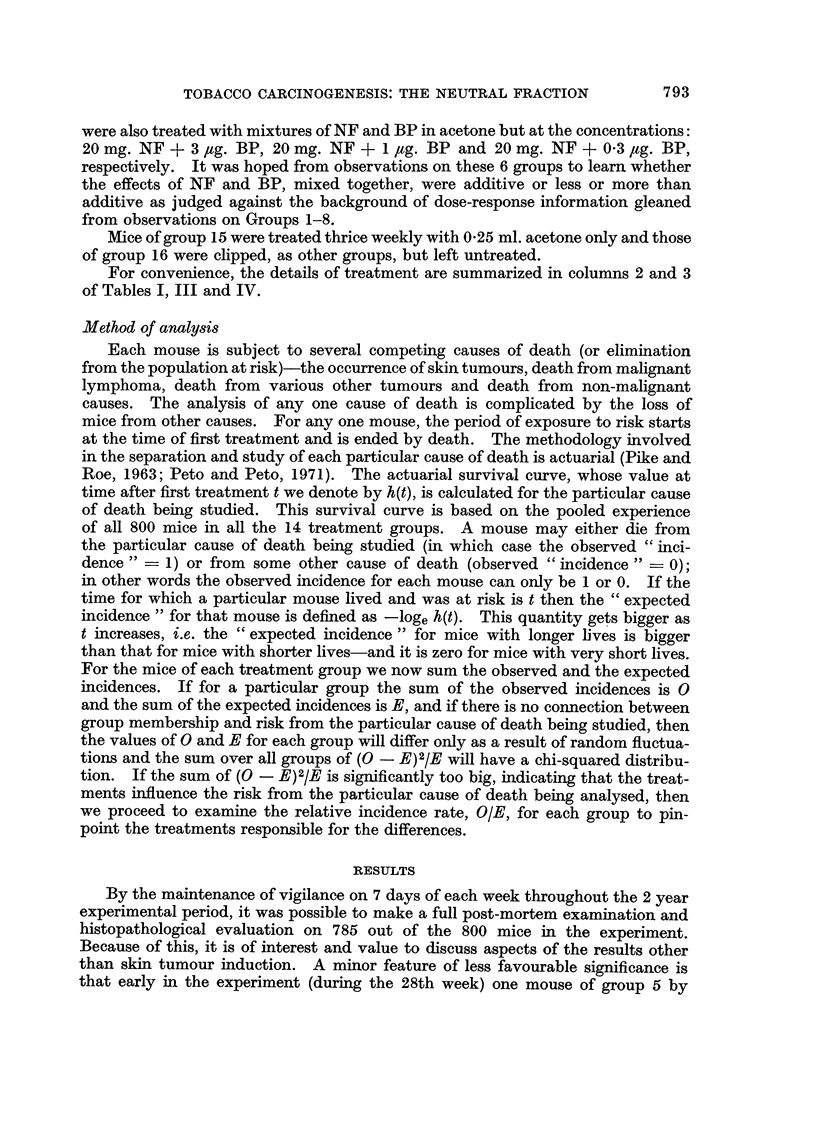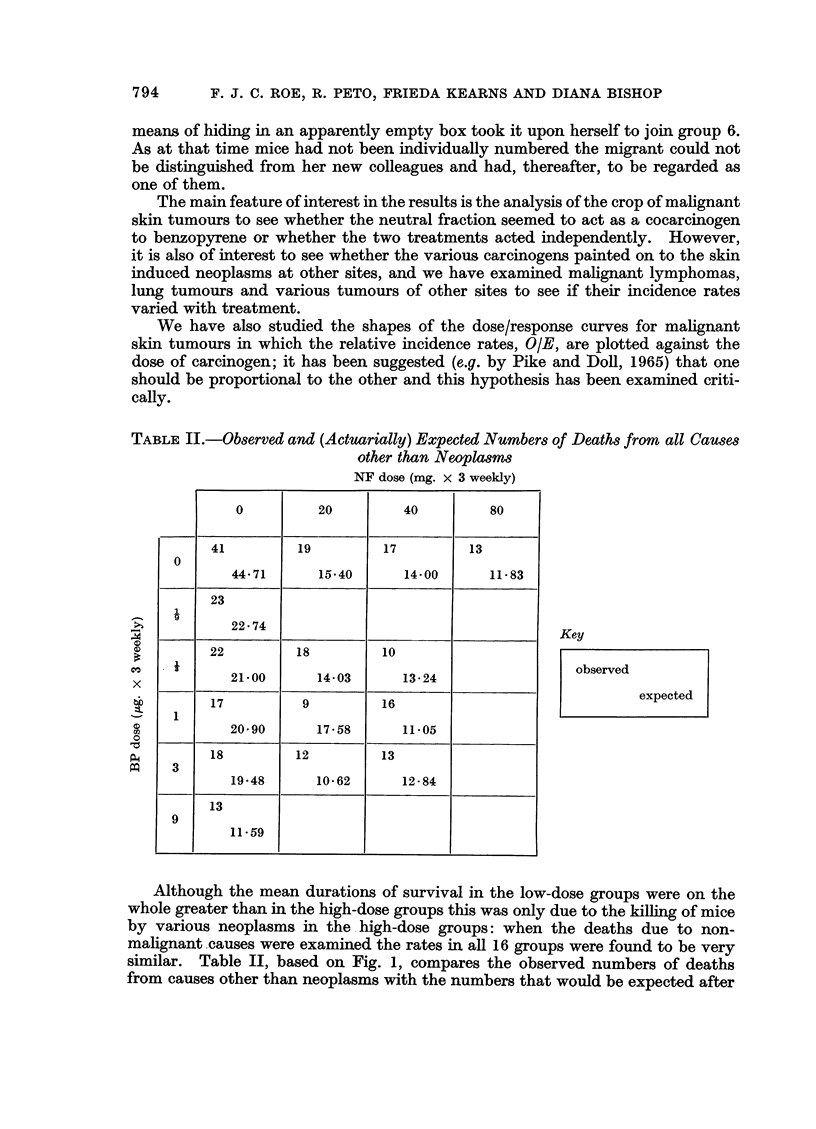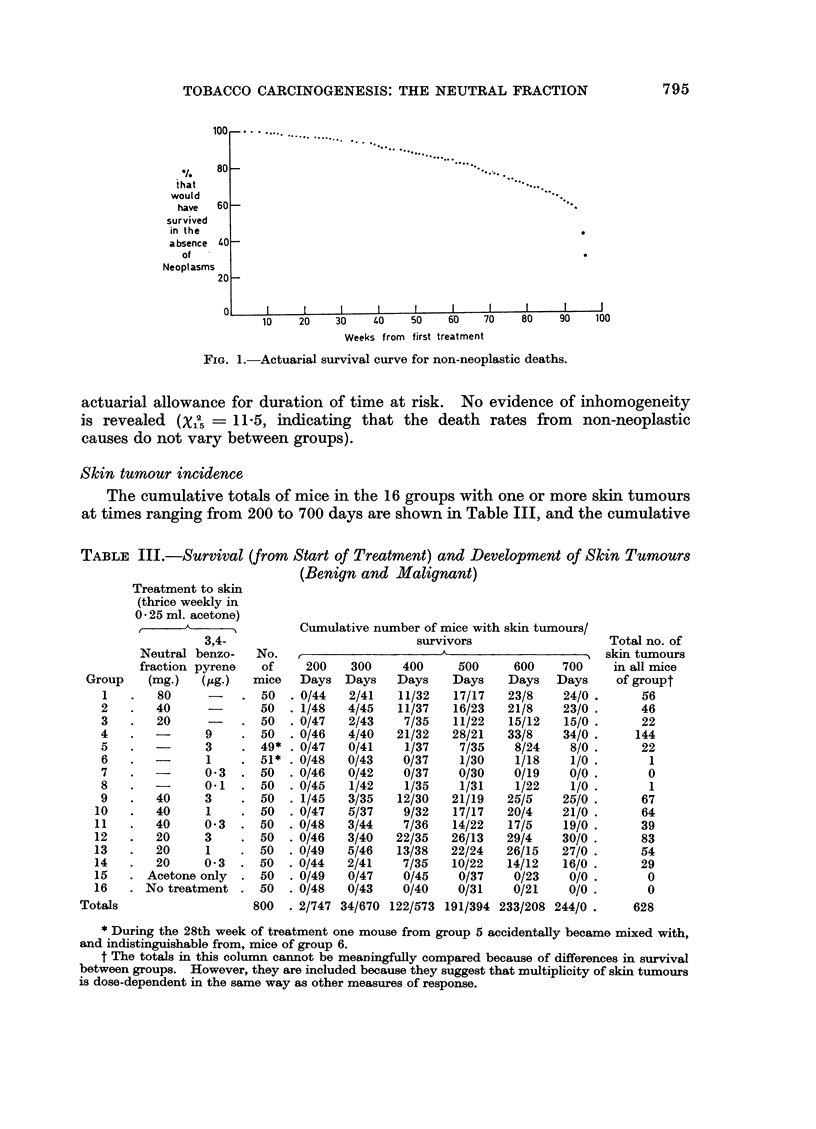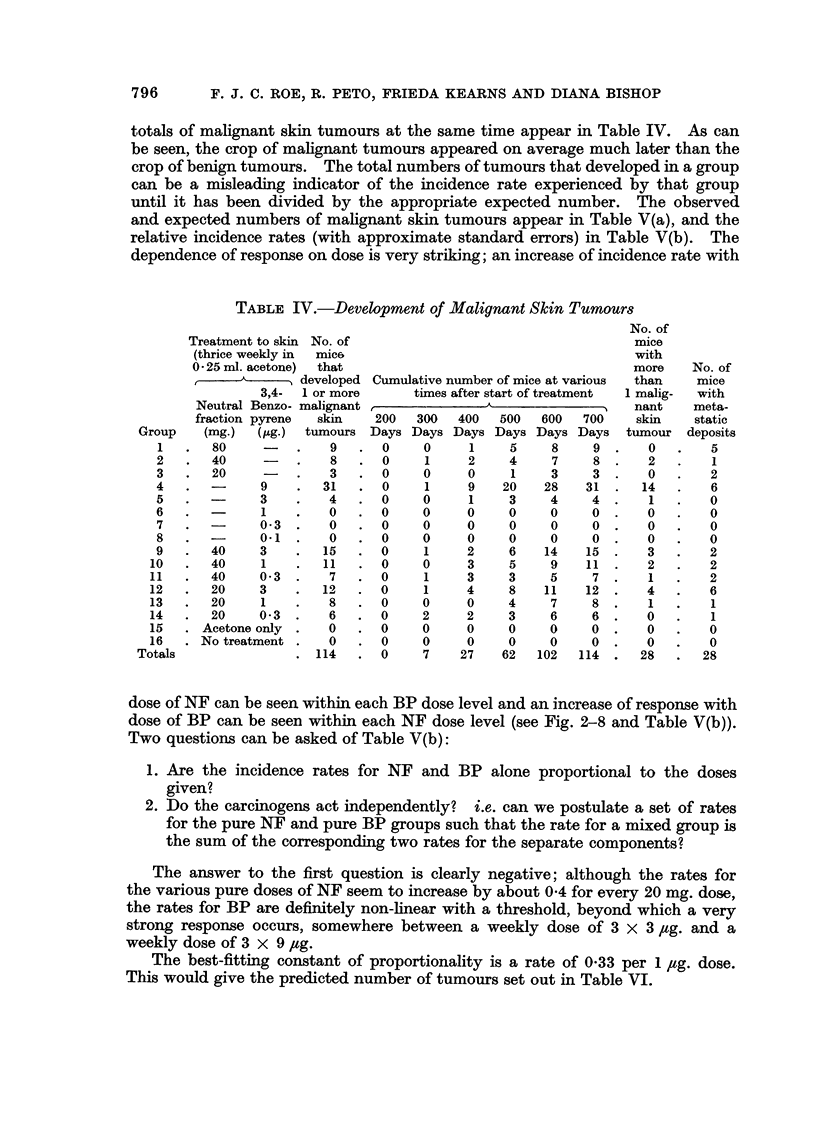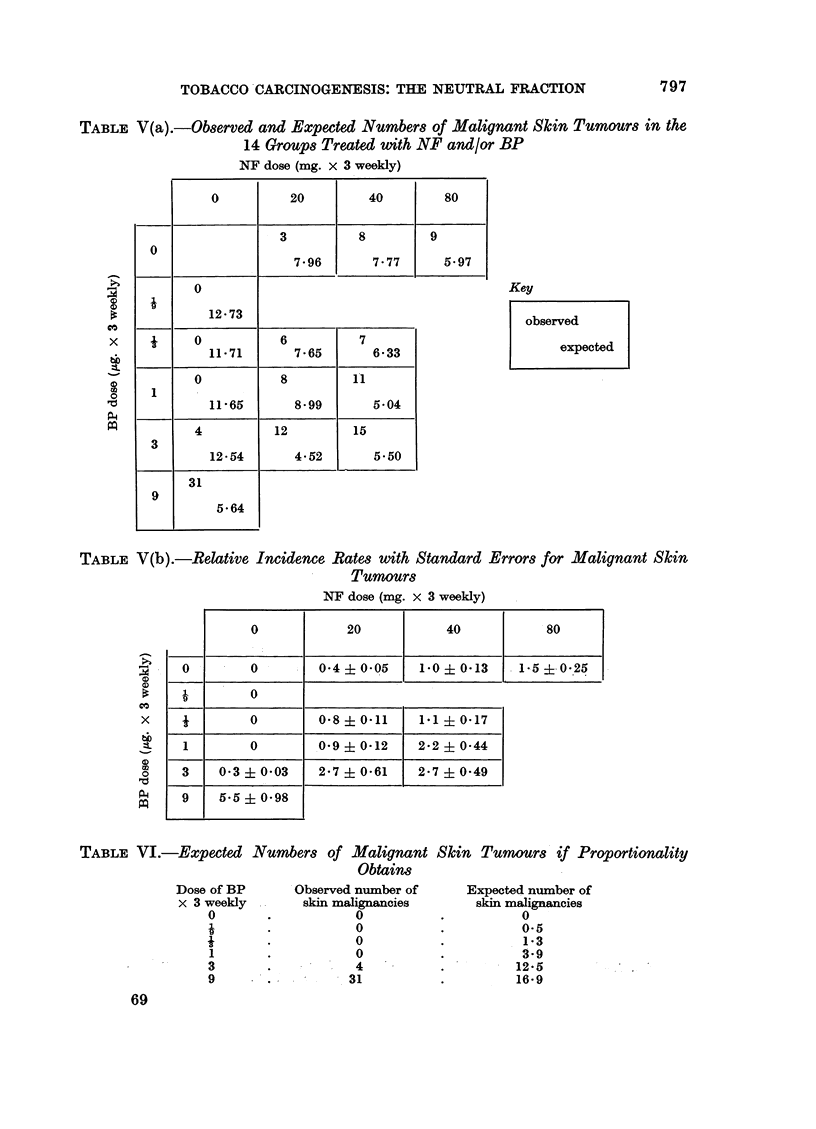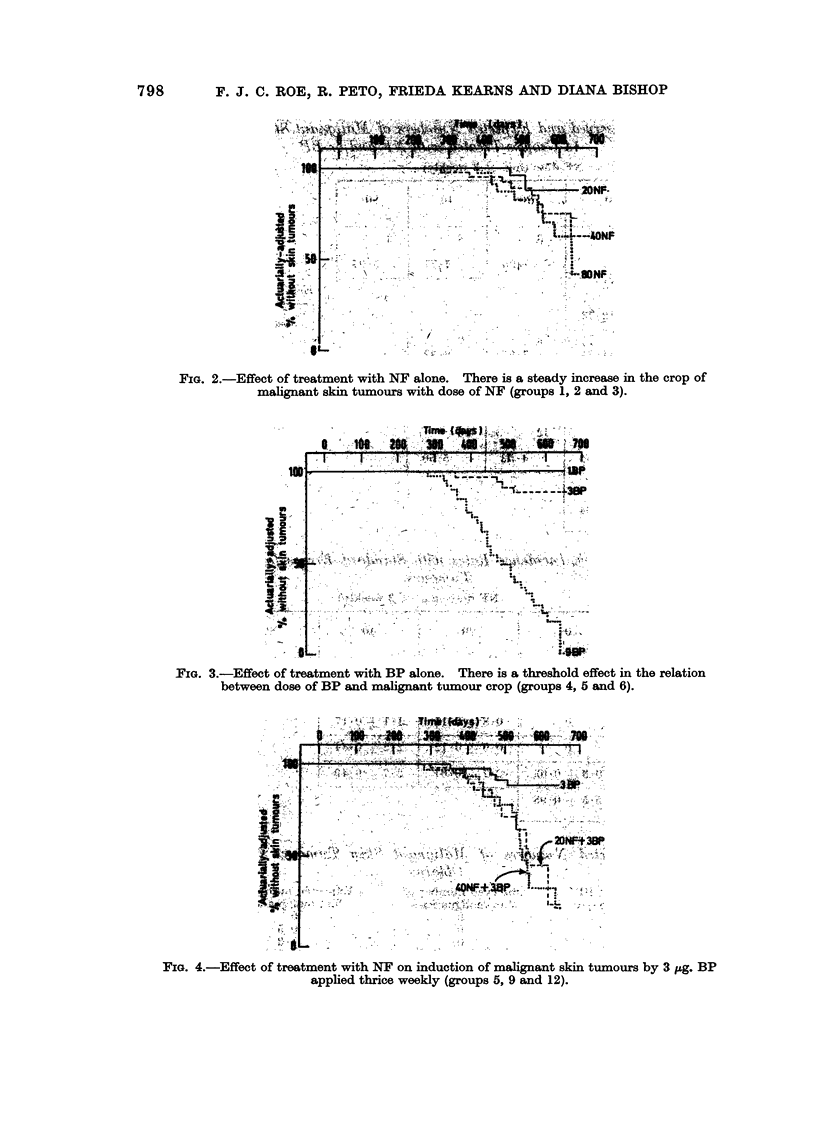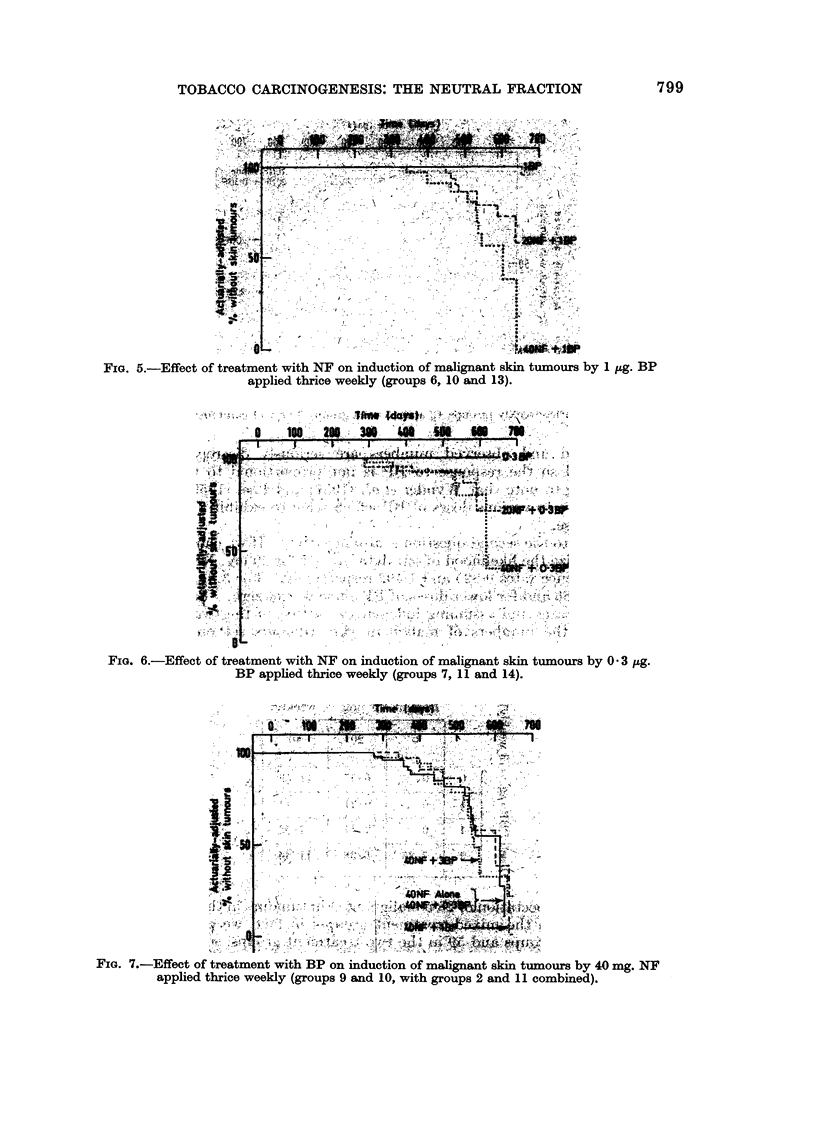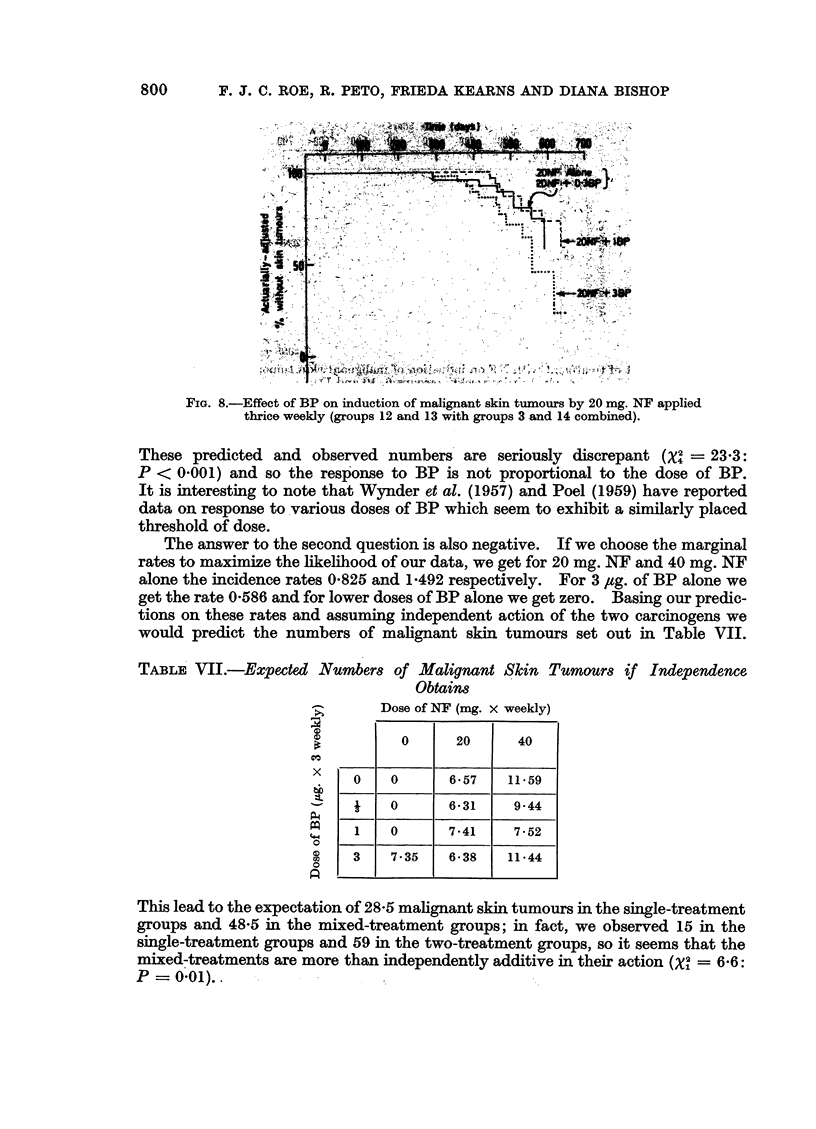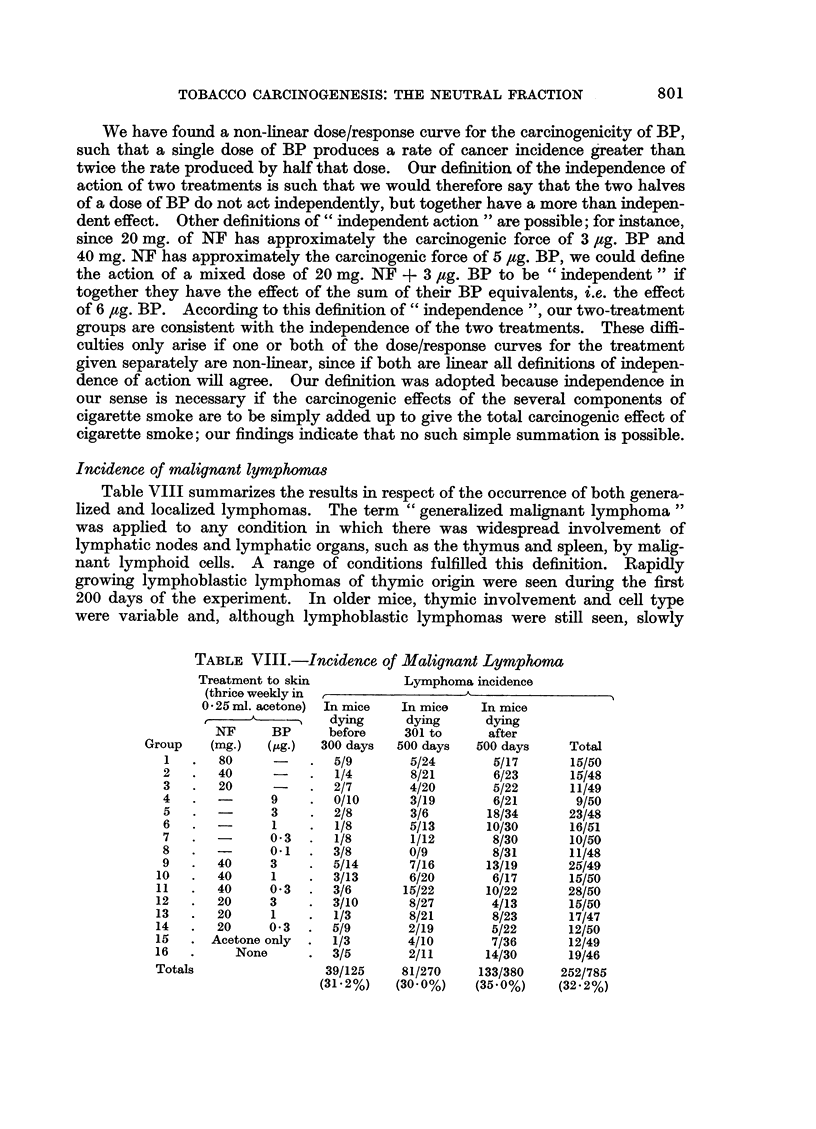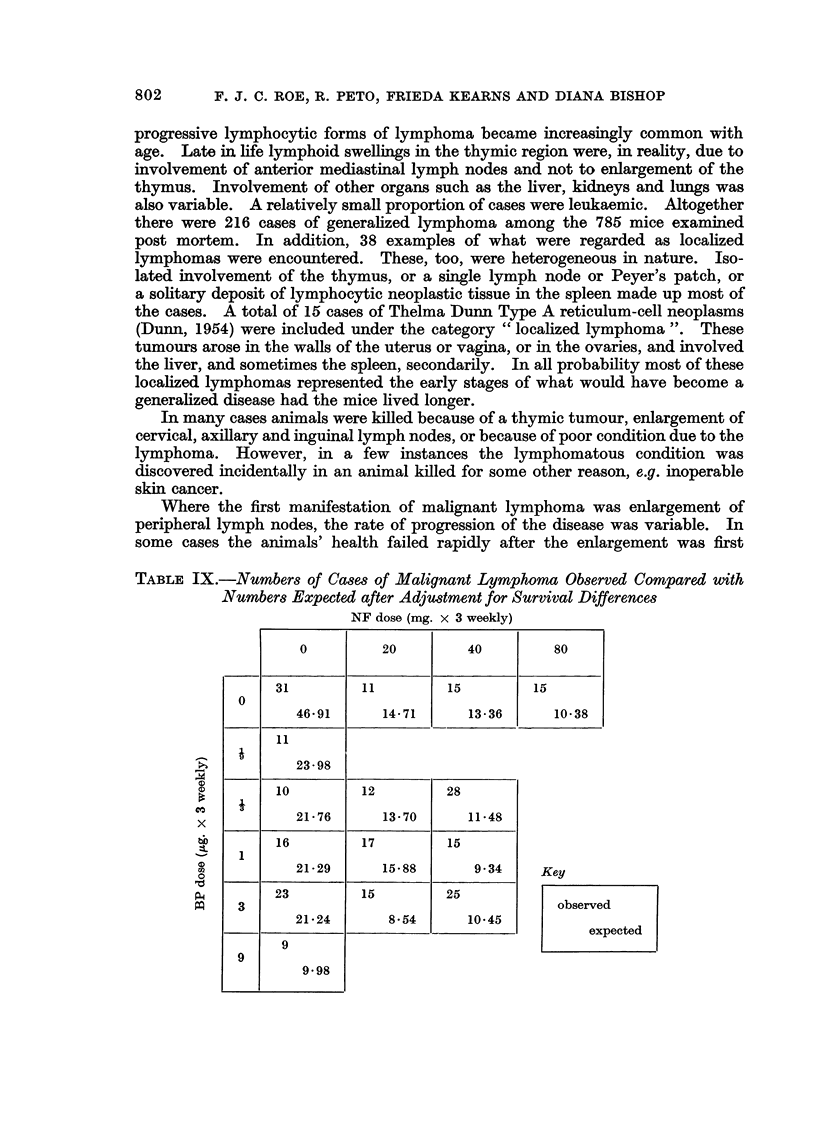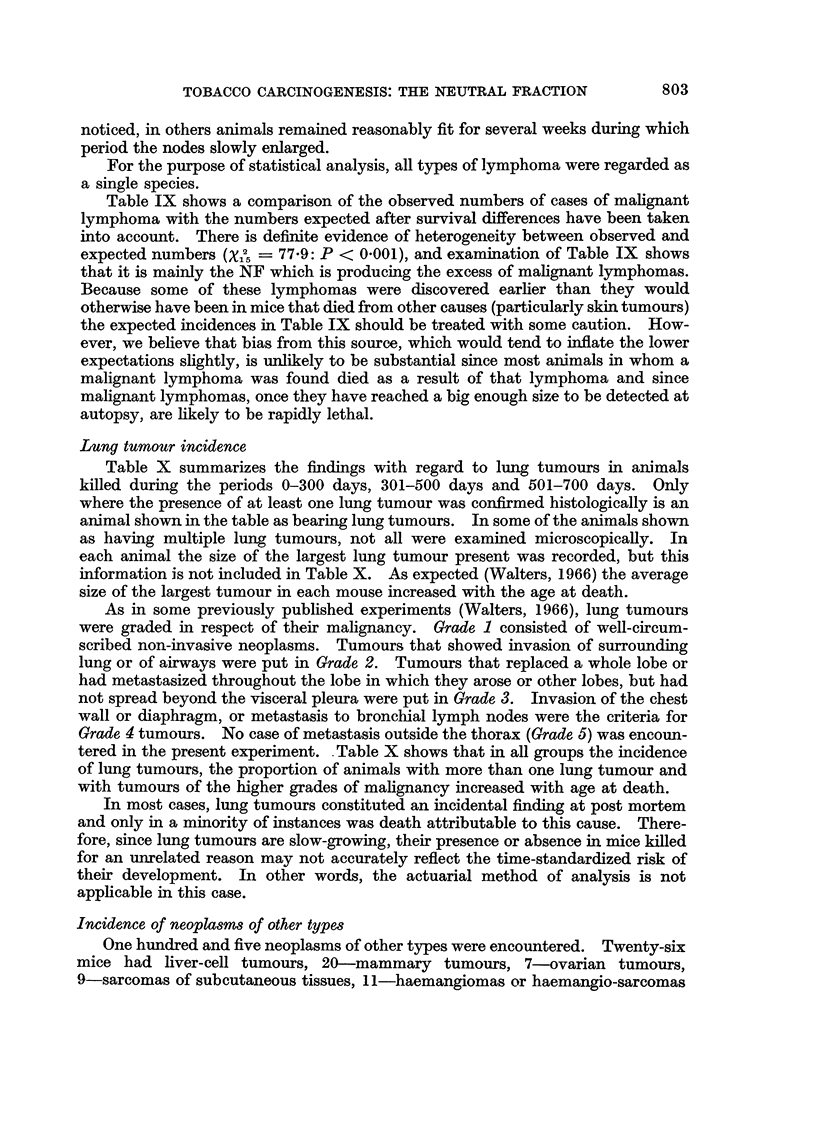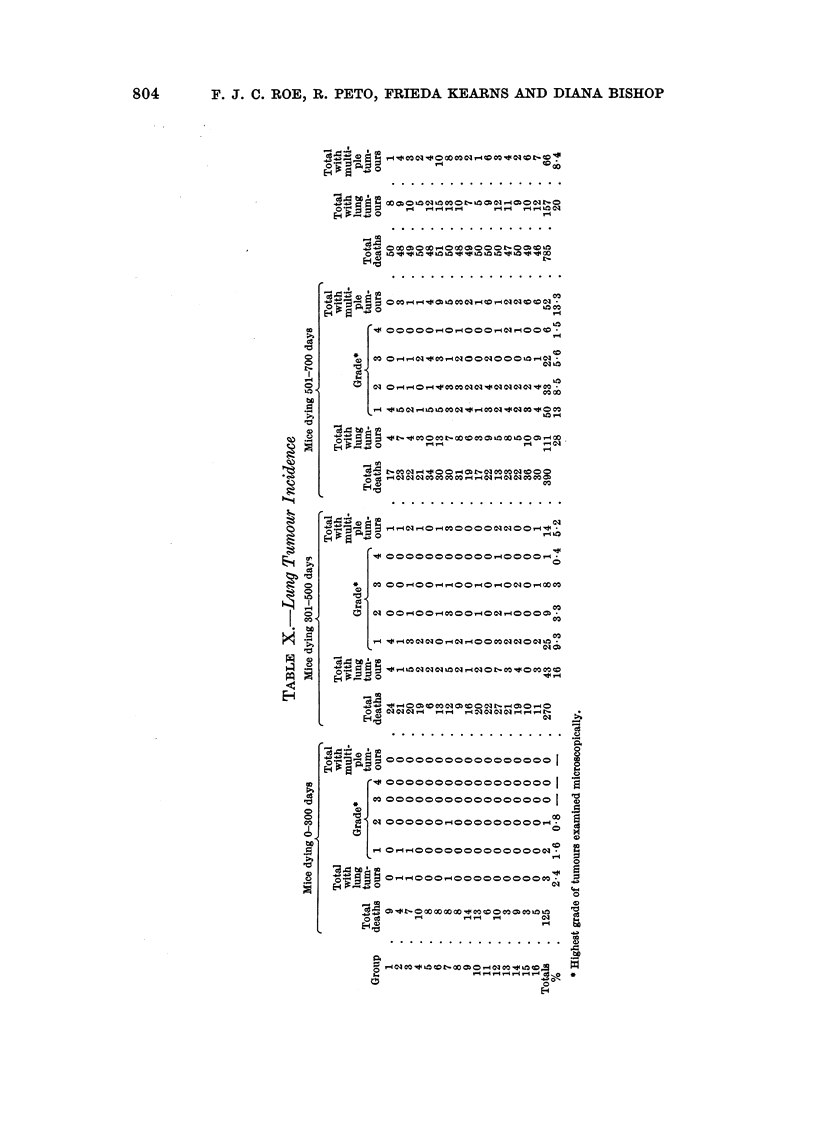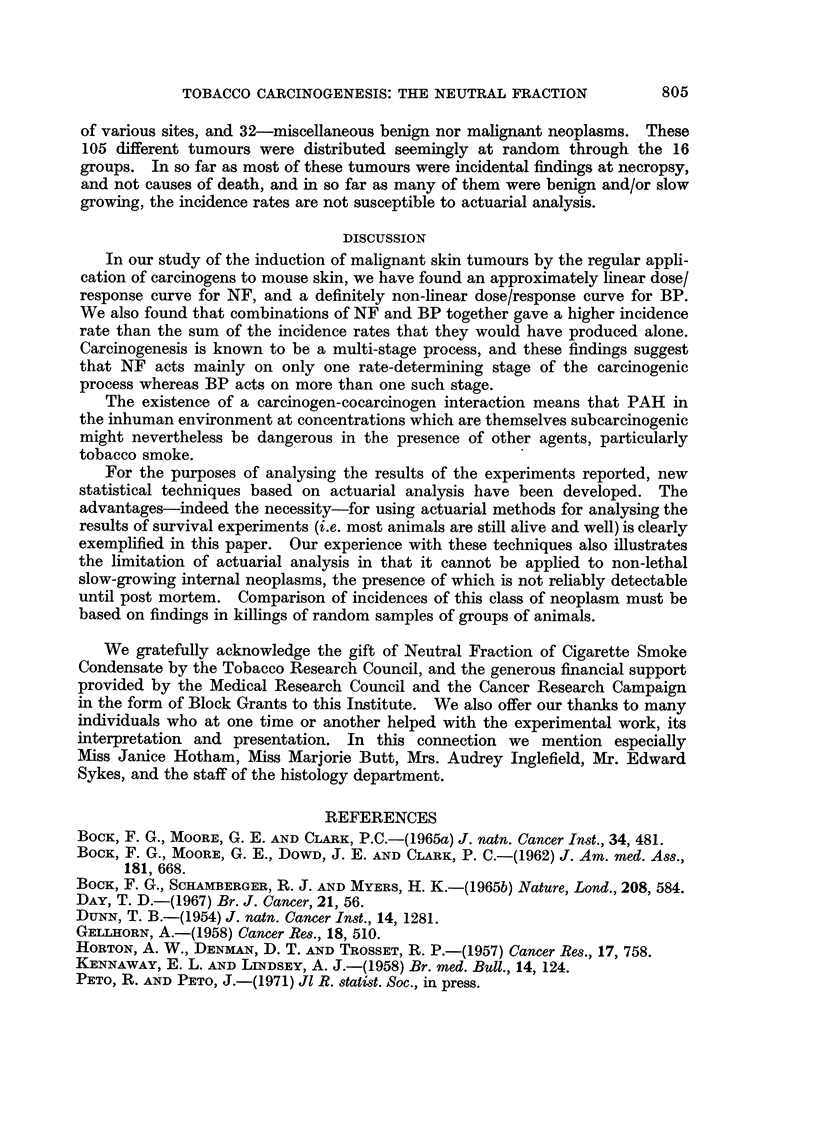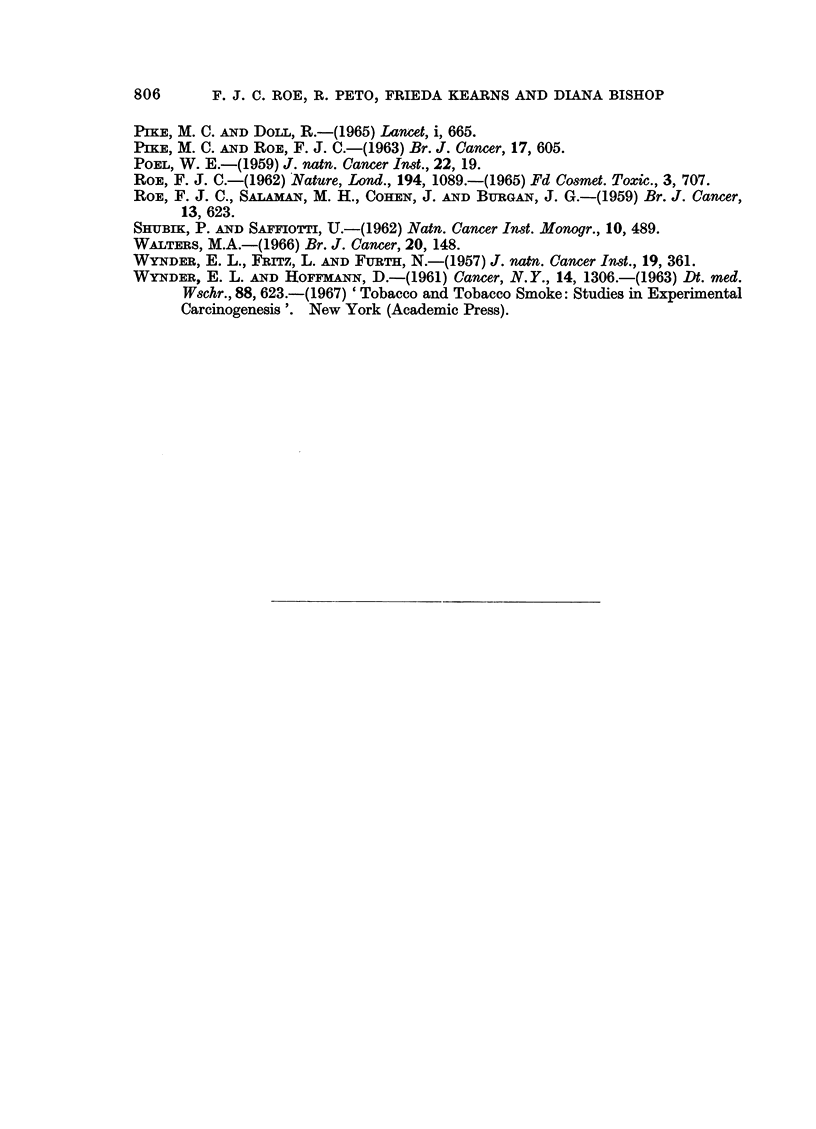# The Mechanism of Carcinogenesis by the Neutral Fraction of Cigarette Smoke Condensate

**DOI:** 10.1038/bjc.1970.94

**Published:** 1970-12

**Authors:** F. J. C. Roe, R. Peto, Frieda Kearns, Diana Bishop

## Abstract

Sixteen groups, each of 50 Swiss female SPF mice, were treated thrice weekly with various combinations of 3,4-benzopyrene (BP) and/or the neutral fraction of cigarette smoke (NF) in acetone applied to the skin. Some groups received one carcinogen, some the other and some a mixture of the two. Skin tumour incidence rates were found to increase both with the dose of NF and with the dose of BP. With BP alone a threshold dose was found beyond which a very heavy incidence rate of malignant skin tumours occurred. After correction of the results for intercurrent deaths it was found that when NF and BP are applied together as a mixture they do not act independently in the production of malignant skin tumours but interact positively. This suggests that some of the components of NF act as cocarcinogens rather than as complete carcinogens. Treatment with NF appeared to increase the incidence of malignant lymphomas. The data were not suitable for deciding whether the various treatments influenced the rates of incidence of internal tumours of other types, for example, lung tumours.


### THE MECHANISM OF CARCINOGENESIS BY THE NEUTRAL FRACTION OF CIGARETTE SMOKE CONDENSATE

F. J. C. ROE, R. PETO, FRIEDA KEARNS AND DIANA BISHOP

*From the Chester Beatty Research Institute, London, S.W.3; and the Department of the Regius Professor of Medicine, Radcliffe Infirmary, Oxford*

Received for publication July 31, 1970

**SUMMARY.—Sixteen groups, each of 50 Swiss female SPF mice, were treated thrice weekly with various combinations of 3,4-benzopyrene (BP) and/or the neutral fraction of cigarette smoke (NF) in acetone applied to the skin. Some groups received one carcinogen, some the other and some a mixture of the two. Skin tumour incidence rates were found to increase both with the dose of NF and with the dose of BP. With BP alone a threshold dose was found beyond which a very heavy incidence rate of malignant skin tumours occurred. After correction of the results for intercurrent deaths it was found that when NF and BP are applied together as a mixture they do not act independently in the production of malignant skin tumours but interact positively. This suggests that some of the components of NF act as cocarcinogens rather than as complete carcinogens. Treatment with NF appeared to increase the incidence of malignant lymphomas. The data were not suitable for deciding whether the various treatments influenced the rates of incidence of internal tumours of other types, for example, lung tumours.**

THE repeated application of cigarette smoke condensate to the skin of mice or rabbits may induce benign and malignant epithelial tumours. The type of tobacco, the way it is cut and packed into cigarettes, the conditions of smoking, the freshness of the condensate at the time of its application to the test animals, the dose of condensate applied, and the strain of test animal are amongst many factors that are known to influence the time and rate of appearance of skin tumours. Nevertheless, provided the test species is sufficiently sensitive and the dose of condensate applied is sufficiently high, positive results are obtained despite wide variations in the other conditions mentioned (for review, see Wynder and Hoffmann, 1967).

Tobacco smoke condensate has been chemically fractionated by a number of different methods and the resultant fractions tested separately for carcinogenicity, mainly by the method of repeated application to mouse skin. A consistent finding in these tests, irrespective of the fractionation technique, has been that the major part of the carcinogenic activity of the whole smoke condensate can be accounted for by the activity of a neutral fraction that remains after removal of carboxylic acids, phenols and bases, including the nicotine alkaloids.

In the neutral fraction, several polynuclear aromatic hydrocarbons (PAH), known to be potent carcinogens, have been identified (Kennaway and Lindsey, 1958; Wynder and Hoffmann, 1963), but it is unlikely that the carcinogenic activity of the neutral fraction is solely due to the carcinogenic PAH that have so far been identified in it. Possibly, constituents of the neutral fraction other than PAH contribute to its carcinogenicity, either by acting as complete carcinogens or as co-carcinogens.

Roe *et al.* (1959) showed that the phenolic fraction of tobacco smoke condensate, which on its own has little or no tumorigenic effect, could promote skin tumour formation in mice pretreated with a subcarcinogenic dose of 7,12-dimethylbenz(a)anthracene. They suggested that the carcinogenicity of the whole condensate may be due to the combined effect of carcinogens (probably of the PAH type) and cocarcinogens, such as phenols. Wynder and Hoffmann (1961) confirmed these findings. Later, Bock *et al.* (1962, 1965*a*, 1965*b*) reported that the heptane-soluble components of whole smoke condensate which, according to them, would not include the phenolic constituents, possessed higher carcinogenic activity than did the smoke condensate itself. They felt that their data conflicted with the view that phenols played a role in the carcinogenicity of smoke condensates. More force was given to the suggestion that cocarcinogens in the condensate were of importance by the finding of Roe (1962) that carcinogenicity of a low concentration of 3,4-benzopyrene may be greatly enhanced if tobacco smoke condensate is applied simultaneously. However, neither this finding, nor that of Gellhorn (1958) could be regarded as conclusive evidence of carcinogen–cocarcinogen interaction, since in neither case were there adequate dose-response data for BP alone or for smoke condensate alone.

The discovery that certain straight chain aliphatic compounds, in particular dodecane, may act as cocarcinogens (Horton *et al.*, 1957; Shubik and Saffiotti, 1962) opened up a further possibility, namely that the carcinogenicity of the neutral fraction of smoke condensate for mouse skin may itself be due to the combined action of carcinogens and cocarcinogens. The experiment described in the present paper was designed to investigate this possibility. The actual plan, however, depended on the assumption that, if the neutral fraction of tobacco smoke contains cocarcinogens, their concentration is such that they would enhance the activity not only of carcinogens present in the neutral fraction, but also of further carcinogens added to the neutral fraction. The plan involved an evaluation of the carcinogenicity for mouse-skin of combinations of NF and BP at various concentrations in the light of dose-response data for BP alone and NF alone.

### MATERIALS AND METHODS

### *Mice*

Eight hundred female Swiss mice were kindly supplied to us by Dr. D. G. Davey of Imperial Chemicals Limited. These animals were born and reared under barrier conditions and were free of specified pathogens (SPF) up to the age of 6 weeks when they were transferred to our experimental unit.

The latter consisted of a separate vermin-proof room in which no other animals were kept. However, there were no facilities for sterilization of food or of the wood-shavings provided as bedding. In other words, from the age of 6 weeks onwards the animals were kept under clean conventional conditions.

During the experiment mice were housed in macralon boxes, 10 per box, and provided with cubed Diet 41B (Messrs. Dixon, Ware, Herts.) and water *ad libitum*.

On arrival in our unit, at the age of 6 weeks, all mice were vaccinated with sheep lymph on the tail as a precaution against ectromelia. Mice in each cage were rendered distinguishable from each other by a system of holes and notches punched on the ears—but this was not done until skin tumours began to appear (*i.e.* approximately 30 weeks). Mice were, on average, $9\frac{1}{2}$ weeks old at the start of treatment.

*Neutral Fraction (NF) of cigarette smoke condensate*

This was most kindly provided by Dr. Whitehead, Harrogate Laboratories, Tobacco Research Council. It was prepared by the smoking of standard 70 mm. long and 25·3 mm. circumference cigarettes of the types, and in the proportions, representative of those of the main brands sold on the British market.

The cigarettes were smoked in automatic smoking machines set to collect 25 ml. smoke in 2 seconds once every minute, and to smoke cigarettes down to a butt length of 20 mm. Smoke was condensed in a series of four glass traps cooled by acetone and crushed solid carbon dioxide. The condensate was washed from the traps by acetone at room temperature and the washings were filtered through glass wool (for details, see Day, 1967).

The NF was prepared from the whole smoke condensate as follows: smoke condensate in acetone as derived from the traps was mixed with diethyl ether (redistilled peroxide-free either from which fluorescent material had been removed by treatment with sodium) and 2N hydrochloric acid. After removal of the aqueous layer, and further similar extractions with hydrochloric acid, the ether solution was repeatedly extracted with 3% w/v aqueous potassium hydroxide solution. Following the final extraction, the ether solution was dried with anhydrous magnesium sulphate, filtered, and the ether removed in a rotary evaporator (for full details, see Day, 1967).

Batches of NF were sent at 2-week intervals from Harrogate to this Institute and, apart from a period of up to 6 hours in transit, the material was stored at 0–4° C. between production and use. The interval between production and use was kept as short as possible and rarely exceeded 3 weeks.

3,4-Benzopyrene (BP) was obtained from L. Light and Company, and Acetone (Analar Grade) from Messrs. Hopkins and Williams.

*Preparation of solutions for application to mice*

Treatment consisted of the repeated application to the dorsal skin of acetone solutions which contained NF alone, BP alone, or both NF and BP in a variety of combinations. Once every 2 weeks, solutions of NF were prepared by weighing appropriate amounts of NF into ground-glass-stoppered amber bottles and adding acetone so that the dose of NF required for each animal in a particular experimental group was contained in 0·25 ml. Measured amounts of a standard BP/acetone solution were added to the NF before addition of acetone where appropriate. Solutions of BP at various concentrations were prepared by dilution of the standard solution with further acetone. All solutions were kept in a refrigerator at 0–4° C. until used.

*Application of solutions to mice*

At the start of the experiment and thereafter at approximately weekly intervals hair was removed from the entire dorsum of each mouse by electric clippers lubricated with liquid paraffin BPC.

Solutions were delivered on to the clipped area from calibrated pipettes in such a way that they spread out over more or less the whole of the clipped area.

*Examination of mice for skin tumour development and other lesions*

Mice were carefully examined at fortnightly intervals for the development of skin tumours, and on each occasion the number of tumours, the site and size of each tumour, and their apparent benignity or malignancy were recorded.

In addition, records were made both at the times of the fortnightly examinations and at other times of the state of general health of individual mice and of other conditions.

Until the end of the 93rd week of painting, mice were kept under treatment and observation unless they were noticeably sick and thought unlikely to survive for more than a few days. Such mice were killed. The experiment was terminated at 93 weeks and all the animals killed and examined post mortem during the following 3 weeks.

*Removal of malignant skin tumours at operation*

Skin tumours considered, on the basis of their macroscopic appearances, to be malignant were removed under ether anaesthesia. It was thought that after operation such animals could be returned to the experiment and be at risk of developing further skin tumours, including further malignant tumours; however, because of difficulties in interpretation, it was later decided to ignore information with regard to skin tumours obtained subsequent to the removal of a malignant tumour by biopsy.

Ninety-four such operations were carried out on 73 different mice. In 77 instances the macroscopic diagnosis was confirmed histologically, in a further 9 the lesion was regarded as " probably malignant " (see below), and in the remaining 8 instances the tumour removed was histologically benign (see Table I).

TABLE I.—*Accuracy of Macroscopic Diagnosis of Malignancy in Relation to Skin Tumours*

| Group | Treatment to skin (thrice weekly) NF (mg.) | BP (μg.) | No. of mice from which presumed malignant skin tumours were removed surgically | No. of mice subjected to more than one such operation | Total number of skin biopsy operations | Histological report: Malignancy confirmed | Tumour designated " probably malignant " | Tumour designated " benign " |
|---|---|---|---|---|---|---|---|---|
| 1 | 80 | — | 5 | 1 | 6 | 5 | 1 | 0 |
| 2 | 40 | — | 4 | 1 | 5 | 4 | 0 | 1 |
| 3 | 20 | — | 4 | 2 | 6 | 4 | 1 | 1 |
| 4 | — | 9 | 22 | 9 | 35 | 28 | 5 | 2 |
| 5 | — | 3 | 4 | 1 | 5 | 4 | 0 | 1 |
| 6 | — | 1 | 0 | | | | | |
| 7 | — | 0·3 | 0 | | | | | |
| 8 | — | 0·1 | 0 | | | | | |
| 9 | 40 | 3 | 9 | 0 | 9 | 8 | 1 | 0 |
| 10 | 40 | 1 | 4 | 0 | 4 | 4 | 0 | 0 |
| 11 | 40 | 0·3 | 5 | 1 | 6 | 5 | 0 | 1 |
| 12 | 20 | 3 | 10 | 1 | 12 | 10 | 1 | 1 |
| 13 | 20 | 1 | 4 | 0 | 4 | 3 | 0 | 1 |
| 14 | 20 | 0·3 | 2 | 0 | 2 | 2 | 0 | 0 |
| 15 | Acetone only | | 0 | | | | | |
| 16 | None | | 0 | | | | | |
| Totals | | | 73 | 16 | 94 | 77 | 9 | 8 |

Seven other animals were operated upon during the experiment: 3 to confirm a macroscopic diagnosis of inflammatory ulceration of the skin, 1 to confirm a diagnosis of malignant lymphoma, and 3 to remove neoplasms other than epitheliomas—a haemangiosarcoma from the tail, a subcutaneous sarcoma, and a mammary adenocarcinoma.

*Pathological observations*

All except 15 of the 800 mice were subjected to systemic post mortem examination (Roe, 1965). In most cases mice were killed when seen to be sick, but approximately 10% were found dead and of these 14 were too decomposed for meaningful autopsy. One mouse was lost.

All skin tumours thought to be malignant or possibly malignant, all lesions of tissues other than the skin thought to be neoplastic, and a variety of apparently non-neoplastic lesions were submitted to histological examination. Tissues were fixed in Bouin's solution, cut at 5 $\mu$ and stained with haematoxylin and eosin. Various other stains were used where indicated.

*Histopathological criteria for malignancy of skin tumours*

Invasion or penetration of the panniculus carnosus muscle was taken as the main criterion of malignancy. Lesions that appeared cytologically malignant, or showed invasion of the dermal collagen, were classified as " probably malignant " if they had not reached the level of the panniculus muscle. In practice only a few lesions fell into this category and for the purposes of evaluating the present experiment they were regarded as benign.

Since a skin tumour grows from a microscopic lesion to a large mass over a period of some weeks, it was necessary to define the " time of occurrence " of a skin tumour in such a way that observer variability was reduced to a minimum. A malignant tumour was defined to " occur " when it first reached a diameter of 10 mm. Experience has shown that, although a mouse normally lives for up to several weeks after it first has a 10 mm. malignant tumour, it is uncommon for a tumour of this size, if it has the macroscopic characteristics of malignancy, not to show invasion of the panniculus muscle. A benign tumour was defined as any lump which persisted with a diameter of 2 mm. or more for 2 weeks or more; its " time of occurrence " was the second week.

*Design of experiment*

Mice were allocated non-selectively to 16 equivalent groups, each of 50. Groups 1, 2 and 3 were given, respectively, 80 mg., 40 mg. and 20 mg. NF in 0·25 ml. acetone thrice weekly by application to the dorsal skin. It was hoped from the results obtained in these 3 groups to obtain information on the relation between skin tumour response and dose of NF.

Groups 4–8 were given, respectively, thrice weekly applications of 9 $\mu$g., 3 $\mu$g., 1 $\mu$g., 0·3 $\mu$g. and 0·1 $\mu$g. BP in 0·25 ml. acetone to the dorsal skin. From the results obtained in these 5 groups information on the relation between skin tumour response and dose of BP was hoped for.

Groups 9–11 were given, respectively, the following mixtures of NF and BP: 40 mg. NF + 3 $\mu$g. BP, 40 mg. NF + 1 $\mu$g. BP and 40 mg. NF + 0·3 $\mu$g. BP. These mixtures were applied thrice weekly in 0·25 ml. acetone. Groups 12–14

were also treated with mixtures of NF and BP in acetone but at the concentrations: 20 mg. NF + 3 $\mu$g. BP, 20 mg. NF + 1 $\mu$g. BP and 20 mg. NF + 0·3 $\mu$g. BP, respectively. It was hoped from observations on these 6 groups to learn whether the effects of NF and BP, mixed together, were additive or less or more than additive as judged against the background of dose-response information gleaned from observations on Groups 1–8.

Mice of group 15 were treated thrice weekly with 0·25 ml. acetone only and those of group 16 were clipped, as other groups, but left untreated.

For convenience, the details of treatment are summarized in columns 2 and 3 of Tables I, III and IV.

### *Method of analysis*

Each mouse is subject to several competing causes of death (or elimination from the population at risk)—the occurrence of skin tumours, death from malignant lymphoma, death from various other tumours and death from non-malignant causes. The analysis of any one cause of death is complicated by the loss of mice from other causes. For any one mouse, the period of exposure to risk starts at the time of first treatment and is ended by death. The methodology involved in the separation and study of each particular cause of death is actuarial (Pike and Roe, 1963; Peto and Peto, 1971). The actuarial survival curve, whose value at time after first treatment $t$ we denote by $h(t)$, is calculated for the particular cause of death being studied. This survival curve is based on the pooled experience of all 800 mice in all the 14 treatment groups. A mouse may either die from the particular cause of death being studied (in which case the observed " incidence " = 1) or from some other cause of death (observed " incidence " = 0); in other words the observed incidence for each mouse can only be 1 or 0. If the time for which a particular mouse lived and was at risk is $t$ then the " expected incidence " for that mouse is defined as $-\log_e h(t)$. This quantity gets bigger as $t$ increases, *i.e.* the " expected incidence " for mice with longer lives is bigger than that for mice with shorter lives—and it is zero for mice with very short lives. For the mice of each treatment group we now sum the observed and the expected incidences. If for a particular group the sum of the observed incidences is $O$ and the sum of the expected incidences is $E$, and if there is no connection between group membership and risk from the particular cause of death being studied, then the values of $O$ and $E$ for each group will differ only as a result of random fluctuations and the sum over all groups of $(O - E)^2/E$ will have a chi-squared distribution. If the sum of $(O - E)^2/E$ is significantly too big, indicating that the treatments influence the risk from the particular cause of death being analysed, then we proceed to examine the relative incidence rate, $O/E$, for each group to pinpoint the treatments responsible for the differences.

### RESULTS

By the maintenance of vigilance on 7 days of each week throughout the 2 year experimental period, it was possible to make a full post-mortem examination and histopathological evaluation on 785 out of the 800 mice in the experiment. Because of this, it is of interest and value to discuss aspects of the results other than skin tumour induction. A minor feature of less favourable significance is that early in the experiment (during the 28th week) one mouse of group 5 by means of hiding in an apparently empty box took it upon herself to join group 6. As at that time mice had not been individually numbered the migrant could not be distinguished from her new colleagues and had, thereafter, to be regarded as one of them.

The main feature of interest in the results is the analysis of the crop of malignant skin tumours to see whether the neutral fraction seemed to act as a cocarcinogen to benzopyrene or whether the two treatments acted independently. However, it is also of interest to see whether the various carcinogens painted on to the skin induced neoplasms at other sites, and we have examined malignant lymphomas, lung tumours and various tumours of other sites to see if their incidence rates varied with treatment.

We have also studied the shapes of the dose/response curves for malignant skin tumours in which the relative incidence rates, $O/E$, are plotted against the dose of carcinogen; it has been suggested (*e.g.* by Pike and Doll, 1965) that one should be proportional to the other and this hypothesis has been examined critically.

TABLE II.—*Observed and (Actuarially) Expected Numbers of Deaths from all Causes other than Neoplasms*

NF dose (mg. × 3 weekly)

| BP dose (μg. × 3 weekly) | 0 | 20 | 40 | 80 |
|---|---|---|---|---|
| 0 | 41 / 44·71 | 19 / 15·40 | 17 / 14·00 | 13 / 11·83 |
| 1/9 | 23 / 22·74 | | | |
| 1/3 | 22 / 21·00 | 18 / 14·03 | 10 / 13·24 | |
| 1 | 17 / 20·90 | 9 / 17·58 | 16 / 11·05 | |
| 3 | 18 / 19·48 | 12 / 10·62 | 13 / 12·84 | |
| 9 | 13 / 11·59 | | | |

*Key*: observed / expected

Although the mean durations of survival in the low-dose groups were on the whole greater than in the high-dose groups this was only due to the killing of mice by various neoplasms in the high-dose groups: when the deaths due to non-malignant causes were examined the rates in all 16 groups were found to be very similar. Table II, based on Fig. 1, compares the observed numbers of deaths from causes other than neoplasms with the numbers that would be expected after

FIG. 1.—Actuarial survival curve for non-neoplastic deaths.

actuarial allowance for duration of time at risk. No evidence of inhomogeneity is revealed ($\chi^2_{15} = 11{\cdot}5$, indicating that the death rates from non-neoplastic causes do not vary between groups).

*Skin tumour incidence*

The cumulative totals of mice in the 16 groups with one or more skin tumours at times ranging from 200 to 700 days are shown in Table III, and the cumulative

TABLE III.—*Survival (from Start of Treatment) and Development of Skin Tumours (Benign and Malignant)*

| Group | Treatment to skin (thrice weekly in 0·25 ml. acetone): Neutral fraction (mg.) | Treatment to skin: 3,4-benzopyrene (µg.) | No. of mice | Cumulative number of mice with skin tumours/survivors: 200 Days | 300 Days | 400 Days | 500 Days | 600 Days | 700 Days | Total no. of skin tumours in all mice of group† |
|---|---|---|---|---|---|---|---|---|---|---|
| 1 | 80 | — | 50 | 0/44 | 2/41 | 11/32 | 17/17 | 23/8 | 24/0 | 56 |
| 2 | 40 | — | 50 | 1/48 | 4/45 | 11/37 | 16/23 | 21/8 | 23/0 | 46 |
| 3 | 20 | — | 50 | 0/47 | 2/43 | 7/35 | 11/22 | 15/12 | 15/0 | 22 |
| 4 | — | 9 | 50 | 0/46 | 4/40 | 21/32 | 28/21 | 33/8 | 34/0 | 144 |
| 5 | — | 3 | 49* | 0/47 | 0/41 | 1/37 | 7/35 | 8/24 | 8/0 | 22 |
| 6 | — | 1 | 51* | 0/48 | 0/43 | 0/37 | 1/30 | 1/18 | 1/0 | 1 |
| 7 | — | 0·3 | 50 | 0/46 | 0/42 | 0/37 | 0/30 | 0/19 | 0/0 | 0 |
| 8 | — | 0·1 | 50 | 0/45 | 1/42 | 1/35 | 1/31 | 1/22 | 1/0 | 1 |
| 9 | 40 | 3 | 50 | 1/45 | 3/35 | 12/30 | 21/19 | 25/5 | 25/0 | 67 |
| 10 | 40 | 1 | 50 | 0/47 | 5/37 | 9/32 | 17/17 | 20/4 | 21/0 | 64 |
| 11 | 40 | 0·3 | 50 | 0/48 | 3/44 | 7/36 | 14/22 | 17/5 | 19/0 | 39 |
| 12 | 20 | 3 | 50 | 0/46 | 3/40 | 22/35 | 26/13 | 29/4 | 30/0 | 83 |
| 13 | 20 | 1 | 50 | 0/49 | 5/46 | 13/38 | 22/24 | 26/15 | 27/0 | 54 |
| 14 | 20 | 0·3 | 50 | 0/44 | 2/41 | 7/35 | 10/22 | 14/12 | 16/0 | 29 |
| 15 | Acetone only | | 50 | 0/49 | 0/47 | 0/45 | 0/37 | 0/23 | 0/0 | 0 |
| 16 | No treatment | | 50 | 0/48 | 0/43 | 0/40 | 0/31 | 0/21 | 0/0 | 0 |
| Totals | | | 800 | 2/747 | 34/670 | 122/573 | 191/394 | 233/208 | 244/0 | 628 |

* During the 28th week of treatment one mouse from group 5 accidentally became mixed with, and indistinguishable from, mice of group 6.

† The totals in this column cannot be meaningfully compared because of differences in survival between groups. However, they are included because they suggest that multiplicity of skin tumours is dose-dependent in the same way as other measures of response.

totals of malignant skin tumours at the same time appear in Table IV. As can be seen, the crop of malignant tumours appeared on average much later than the crop of benign tumours. The total numbers of tumours that developed in a group can be a misleading indicator of the incidence rate experienced by that group until it has been divided by the appropriate expected number. The observed and expected numbers of malignant skin tumours appear in Table V(a), and the relative incidence rates (with approximate standard errors) in Table V(b). The dependence of response on dose is very striking; an increase of incidence rate with

TABLE IV.—*Development of Malignant Skin Tumours*

| Group | Treatment to skin (thrice weekly in 0·25 ml. acetone): Neutral fraction (mg.) | 3,4-Benzopyrene (µg.) | No. of mice that developed 1 or more malignant skin tumours | Cumulative number of mice at various times after start of treatment: 200 Days | 300 Days | 400 Days | 500 Days | 600 Days | 700 Days | No. of mice with more than 1 malignant skin tumour | No. of mice with metastatic deposits |
|---|---|---|---|---|---|---|---|---|---|---|---|
| 1 | 80 | — | 9 | 0 | 0 | 1 | 5 | 8 | 9 | 0 | 5 |
| 2 | 40 | — | 8 | 0 | 1 | 2 | 4 | 7 | 8 | 2 | 1 |
| 3 | 20 | — | 3 | 0 | 0 | 0 | 1 | 3 | 3 | 0 | 2 |
| 4 | — | 9 | 31 | 0 | 1 | 9 | 20 | 28 | 31 | 14 | 6 |
| 5 | — | 3 | 4 | 0 | 0 | 1 | 3 | 4 | 4 | 1 | 0 |
| 6 | — | 1 | 0 | 0 | 0 | 0 | 0 | 0 | 0 | 0 | 0 |
| 7 | — | 0·3 | 0 | 0 | 0 | 0 | 0 | 0 | 0 | 0 | 0 |
| 8 | — | 0·1 | 0 | 0 | 0 | 0 | 0 | 0 | 0 | 0 | 0 |
| 9 | 40 | 3 | 15 | 0 | 1 | 2 | 6 | 14 | 15 | 3 | 2 |
| 10 | 40 | 1 | 11 | 0 | 0 | 3 | 5 | 9 | 11 | 2 | 2 |
| 11 | 40 | 0·3 | 7 | 0 | 1 | 3 | 3 | 5 | 7 | 1 | 2 |
| 12 | 20 | 3 | 12 | 0 | 1 | 4 | 8 | 11 | 12 | 4 | 6 |
| 13 | 20 | 1 | 8 | 0 | 0 | 0 | 4 | 7 | 8 | 1 | 1 |
| 14 | 20 | 0·3 | 6 | 0 | 2 | 2 | 3 | 6 | 6 | 0 | 1 |
| 15 | Acetone only | | 0 | 0 | 0 | 0 | 0 | 0 | 0 | 0 | 0 |
| 16 | No treatment | | 0 | 0 | 0 | 0 | 0 | 0 | 0 | 0 | 0 |
| Totals | | | 114 | 0 | 7 | 27 | 62 | 102 | 114 | 28 | 28 |

dose of NF can be seen within each BP dose level and an increase of response with dose of BP can be seen within each NF dose level (see Fig. 2–8 and Table V(b)). Two questions can be asked of Table V(b):

1. Are the incidence rates for NF and BP alone proportional to the doses given?
2. Do the carcinogens act independently? *i.e.* can we postulate a set of rates for the pure NF and pure BP groups such that the rate for a mixed group is the sum of the corresponding two rates for the separate components?

The answer to the first question is clearly negative; although the rates for the various pure doses of NF seem to increase by about 0·4 for every 20 mg. dose, the rates for BP are definitely non-linear with a threshold, beyond which a very strong response occurs, somewhere between a weekly dose of $3 \times 3$ µg. and a weekly dose of $3 \times 9$ µg.

The best-fitting constant of proportionality is a rate of 0·33 per 1 µg. dose. This would give the predicted number of tumours set out in Table VI.

TABLE V(a).—*Observed and Expected Numbers of Malignant Skin Tumours in the 14 Groups Treated with NF and/or BP*

NF dose (mg. × 3 weekly)

| BP dose (µg. × 3 weekly) | 0 | 20 | 40 | 80 |
|---|---|---|---|---|
| 0 | | 3<br>7·96 | 8<br>7·77 | 9<br>5·97 |
| $\frac{1}{9}$ | 0<br>12·73 | | | |
| $\frac{1}{3}$ | 0<br>11·71 | 6<br>7·65 | 7<br>6·33 | |
| 1 | 0<br>11·65 | 8<br>8·99 | 11<br>5·04 | |
| 3 | 4<br>12·54 | 12<br>4·52 | 15<br>5·50 | |
| 9 | 31<br>5·64 | | | |

*Key*

observed
expected

TABLE V(b).—*Relative Incidence Rates with Standard Errors for Malignant Skin Tumours*

NF dose (mg. × 3 weekly)

| BP dose (µg. × 3 weekly) | 0 | 20 | 40 | 80 |
|---|---|---|---|---|
| 0 | 0 | 0·4 ± 0·05 | 1·0 ± 0·13 | 1·5 ± 0·25 |
| $\frac{1}{9}$ | 0 | | | |
| $\frac{1}{3}$ | 0 | 0·8 ± 0·11 | 1·1 ± 0·17 | |
| 1 | 0 | 0·9 ± 0·12 | 2·2 ± 0·44 | |
| 3 | 0·3 ± 0·03 | 2·7 ± 0·61 | 2·7 ± 0·49 | |
| 9 | 5·5 ± 0·98 | | | |

TABLE VI.—*Expected Numbers of Malignant Skin Tumours if Proportionality Obtains*

| Dose of BP × 3 weekly | Observed number of skin malignancies | Expected number of skin malignancies |
|---|---|---|
| 0 | 0 | 0 |
| $\frac{1}{9}$ | 0 | 0·5 |
| $\frac{1}{3}$ | 0 | 1·3 |
| 1 | 0 | 3·9 |
| 3 | 4 | 12·5 |
| 9 | 31 | 16·9 |

FIG. 2.—Effect of treatment with NF alone. There is a steady increase in the crop of malignant skin tumours with dose of NF (groups 1, 2 and 3).

FIG. 3.—Effect of treatment with BP alone. There is a threshold effect in the relation between dose of BP and malignant tumour crop (groups 4, 5 and 6).

FIG. 4.—Effect of treatment with NF on induction of malignant skin tumours by 3 μg. BP applied thrice weekly (groups 5, 9 and 12).

**Fig. 5.**—Effect of treatment with NF on induction of malignant skin tumours by 1 μg. BP applied thrice weekly (groups 6, 10 and 13).

**Fig. 6.**—Effect of treatment with NF on induction of malignant skin tumours by 0·3 μg. BP applied thrice weekly (groups 7, 11 and 14).

**Fig. 7.**—Effect of treatment with BP on induction of malignant skin tumours by 40 mg. NF applied thrice weekly (groups 9 and 10, with groups 2 and 11 combined).

FIG. 8.—Effect of BP on induction of malignant skin tumours by 20 mg. NF applied thrice weekly (groups 12 and 13 with groups 3 and 14 combined).

These predicted and observed numbers are seriously discrepant ($\chi^2_4 = 23{\cdot}3$: $P < 0{\cdot}001$) and so the response to BP is not proportional to the dose of BP. It is interesting to note that Wynder *et al.* (1957) and Poel (1959) have reported data on response to various doses of BP which seem to exhibit a similarly placed threshold of dose.

The answer to the second question is also negative. If we choose the marginal rates to maximize the likelihood of our data, we get for 20 mg. NF and 40 mg. NF alone the incidence rates 0·825 and 1·492 respectively. For 3 $\mu$g. of BP alone we get the rate 0·586 and for lower doses of BP alone we get zero. Basing our predictions on these rates and assuming independent action of the two carcinogens we would predict the numbers of malignant skin tumours set out in Table VII.

TABLE VII.—*Expected Numbers of Malignant Skin Tumours if Independence Obtains*

| Dose of BP ($\mu$g. × 3 weekly) | Dose of NF (mg. × weekly) 0 | 20 | 40 |
|---|---|---|---|
| 0 | 0 | 6·57 | 11·59 |
| $\frac{1}{3}$ | 0 | 6·31 | 9·44 |
| 1 | 0 | 7·41 | 7·52 |
| 3 | 7·35 | 6·38 | 11·44 |

This lead to the expectation of 28·5 malignant skin tumours in the single-treatment groups and 48·5 in the mixed-treatment groups; in fact, we observed 15 in the single-treatment groups and 59 in the two-treatment groups, so it seems that the mixed-treatments are more than independently additive in their action ($\chi^2_1 = 6{\cdot}6$: $P = 0{\cdot}01$).

We have found a non-linear dose/response curve for the carcinogenicity of BP, such that a single dose of BP produces a rate of cancer incidence greater than twice the rate produced by half that dose. Our definition of the independence of action of two treatments is such that we would therefore say that the two halves of a dose of BP do not act independently, but together have a more than independent effect. Other definitions of " independent action " are possible; for instance, since 20 mg. of NF has approximately the carcinogenic force of 3 $\mu$g. BP and 40 mg. NF has approximately the carcinogenic force of 5 $\mu$g. BP, we could define the action of a mixed dose of 20 mg. NF + 3 $\mu$g. BP to be " independent " if together they have the effect of the sum of their BP equivalents, *i.e.* the effect of 6 $\mu$g. BP. According to this definition of " independence ", our two-treatment groups are consistent with the independence of the two treatments. These difficulties only arise if one or both of the dose/response curves for the treatment given separately are non-linear, since if both are linear all definitions of independence of action will agree. Our definition was adopted because independence in our sense is necessary if the carcinogenic effects of the several components of cigarette smoke are to be simply added up to give the total carcinogenic effect of cigarette smoke; our findings indicate that no such simple summation is possible.

*Incidence of malignant lymphomas*

Table VIII summarizes the results in respect of the occurrence of both generalized and localized lymphomas. The term " generalized malignant lymphoma " was applied to any condition in which there was widespread involvement of lymphatic nodes and lymphatic organs, such as the thymus and spleen, by malignant lymphoid cells. A range of conditions fulfilled this definition. Rapidly growing lymphoblastic lymphomas of thymic origin were seen during the first 200 days of the experiment. In older mice, thymic involvement and cell type were variable and, although lymphoblastic lymphomas were still seen, slowly

TABLE VIII.—*Incidence of Malignant Lymphoma*

| Group | Treatment to skin (thrice weekly in 0·25 ml. acetone) NF (mg.) | BP ($\mu$g.) | Lymphoma incidence: In mice dying before 300 days | In mice dying 301 to 500 days | In mice dying after 500 days | Total |
|---|---|---|---|---|---|---|
| 1 | 80 | — | 5/9 | 5/24 | 5/17 | 15/50 |
| 2 | 40 | — | 1/4 | 8/21 | 6/23 | 15/48 |
| 3 | 20 | — | 2/7 | 4/20 | 5/22 | 11/49 |
| 4 | — | 9 | 0/10 | 3/19 | 6/21 | 9/50 |
| 5 | — | 3 | 2/8 | 3/6 | 18/34 | 23/48 |
| 6 | — | 1 | 1/8 | 5/13 | 10/30 | 16/51 |
| 7 | — | 0·3 | 1/8 | 1/12 | 8/30 | 10/50 |
| 8 | — | 0·1 | 3/8 | 0/9 | 8/31 | 11/48 |
| 9 | 40 | 3 | 5/14 | 7/16 | 13/19 | 25/49 |
| 10 | 40 | 1 | 3/13 | 6/20 | 6/17 | 15/50 |
| 11 | 40 | 0·3 | 3/6 | 15/22 | 10/22 | 28/50 |
| 12 | 20 | 3 | 3/10 | 8/27 | 4/13 | 15/50 |
| 13 | 20 | 1 | 1/3 | 8/21 | 8/23 | 17/47 |
| 14 | 20 | 0·3 | 5/9 | 2/19 | 5/22 | 12/50 |
| 15 | Acetone only | | 1/3 | 4/10 | 7/36 | 12/49 |
| 16 | None | | 3/5 | 2/11 | 14/30 | 19/46 |
| Totals | | | 39/125 (31·2%) | 81/270 (30·0%) | 133/380 (35·0%) | 252/785 (32·2%) |

progressive lymphocytic forms of lymphoma became increasingly common with age. Late in life lymphoid swellings in the thymic region were, in reality, due to involvement of anterior mediastinal lymph nodes and not to enlargement of the thymus. Involvement of other organs such as the liver, kidneys and lungs was also variable. A relatively small proportion of cases were leukaemic. Altogether there were 216 cases of generalized lymphoma among the 785 mice examined post mortem. In addition, 38 examples of what were regarded as localized lymphomas were encountered. These, too, were heterogeneous in nature. Isolated involvement of the thymus, or a single lymph node or Peyer's patch, or a solitary deposit of lymphocytic neoplastic tissue in the spleen made up most of the cases. A total of 15 cases of Thelma Dunn Type A reticulum-cell neoplasms (Dunn, 1954) were included under the category " localized lymphoma ". These tumours arose in the walls of the uterus or vagina, or in the ovaries, and involved the liver, and sometimes the spleen, secondarily. In all probability most of these localized lymphomas represented the early stages of what would have become a generalized disease had the mice lived longer.

In many cases animals were killed because of a thymic tumour, enlargement of cervical, axillary and inguinal lymph nodes, or because of poor condition due to the lymphoma. However, in a few instances the lymphomatous condition was discovered incidentally in an animal killed for some other reason, *e.g.* inoperable skin cancer.

Where the first manifestation of malignant lymphoma was enlargement of peripheral lymph nodes, the rate of progression of the disease was variable. In some cases the animals' health failed rapidly after the enlargement was first

TABLE IX.—*Numbers of Cases of Malignant Lymphoma Observed Compared with Numbers Expected after Adjustment for Survival Differences*

| BP dose (μg. × 3 weekly) | NF dose (mg. × 3 weekly) 0 | 20 | 40 | 80 |
|---|---|---|---|---|
| 0 | 31 / 46·91 | 11 / 14·71 | 15 / 13·36 | 15 / 10·38 |
| 1/9 | 11 / 23·98 | | | |
| 1/3 | 10 / 21·76 | 12 / 13·70 | 28 / 11·48 | |
| 1 | 16 / 21·29 | 17 / 15·88 | 15 / 9·34 | |
| 3 | 23 / 21·24 | 15 / 8·54 | 25 / 10·45 | |
| 9 | 9 / 9·98 | | | |

*Key*

observed / expected noticed, in others animals remained reasonably fit for several weeks during which period the nodes slowly enlarged.

For the purpose of statistical analysis, all types of lymphoma were regarded as a single species.

Table IX shows a comparison of the observed numbers of cases of malignant lymphoma with the numbers expected after survival differences have been taken into account. There is definite evidence of heterogeneity between observed and expected numbers ($\chi^2_{15} = 77{\cdot}9$: $P < 0{\cdot}001$), and examination of Table IX shows that it is mainly the NF which is producing the excess of malignant lymphomas. Because some of these lymphomas were discovered earlier than they would otherwise have been in mice that died from other causes (particularly skin tumours) the expected incidences in Table IX should be treated with some caution. However, we believe that bias from this source, which would tend to inflate the lower expectations slightly, is unlikely to be substantial since most animals in whom a malignant lymphoma was found died as a result of that lymphoma and since malignant lymphomas, once they have reached a big enough size to be detected at autopsy, are likely to be rapidly lethal.

*Lung tumour incidence*

Table X summarizes the findings with regard to lung tumours in animals killed during the periods 0–300 days, 301–500 days and 501–700 days. Only where the presence of at least one lung tumour was confirmed histologically is an animal shown in the table as bearing lung tumours. In some of the animals shown as having multiple lung tumours, not all were examined microscopically. In each animal the size of the largest lung tumour present was recorded, but this information is not included in Table X. As expected (Walters, 1966) the average size of the largest tumour in each mouse increased with the age at death.

As in some previously published experiments (Walters, 1966), lung tumours were graded in respect of their malignancy. *Grade 1* consisted of well-circumscribed non-invasive neoplasms. Tumours that showed invasion of surrounding lung or of airways were put in *Grade 2*. Tumours that replaced a whole lobe or had metastasized throughout the lobe in which they arose or other lobes, but had not spread beyond the visceral pleura were put in *Grade 3*. Invasion of the chest wall or diaphragm, or metastasis to bronchial lymph nodes were the criteria for *Grade 4* tumours. No case of metastasis outside the thorax (*Grade 5*) was encountered in the present experiment. Table X shows that in all groups the incidence of lung tumours, the proportion of animals with more than one lung tumour and with tumours of the higher grades of malignancy increased with age at death.

In most cases, lung tumours constituted an incidental finding at post mortem and only in a minority of instances was death attributable to this cause. Therefore, since lung tumours are slow-growing, their presence or absence in mice killed for an unrelated reason may not accurately reflect the time-standardized risk of their development. In other words, the actuarial method of analysis is not applicable in this case.

*Incidence of neoplasms of other types*

One hundred and five neoplasms of other types were encountered. Twenty-six mice had liver-cell tumours, 20—mammary tumours, 7—ovarian tumours, 9—sarcomas of subcutaneous tissues, 11—haemangiomas or haemangio-sarcomas

TABLE X.—*Lung Tumour Incidence*

| | Mice dying 0–300 days | | | | | | | Mice dying 301–500 days | | | | | | | Mice dying 501–700 days | | | | | | | | | |
|---|---|---|---|---|---|---|---|---|---|---|---|---|---|---|---|---|---|---|---|---|---|---|---|---|
| | | | Grade* | | | | | | | Grade* | | | | | | | Grade* | | | | | | | |
| Group | Total deaths | Total with lung tumours | 1 | 2 | 3 | 4 | Total with multiple tumours | Total deaths | Total with lung tumours | 1 | 2 | 3 | 4 | Total with multiple tumours | Total deaths | Total with lung tumours | 1 | 2 | 3 | 4 | Total with multiple tumours | Total deaths | Total with lung tumours | Total with multiple tumours |
| 1 | 9 | 0 | 0 | 0 | 0 | 0 | 0 | 24 | 4 | 4 | 0 | 0 | 0 | 1 | 17 | 4 | 4 | 0 | 0 | 0 | 0 | 50 | 8 | 1 |
| 2 | 4 | 1 | 1 | 0 | 0 | 0 | 0 | 21 | 1 | 1 | 0 | 0 | 0 | 1 | 23 | 7 | 5 | 1 | 1 | 0 | 3 | 48 | 9 | 4 |
| 3 | 7 | 1 | 1 | 0 | 0 | 0 | 0 | 20 | 5 | 3 | 1 | 1 | 0 | 2 | 22 | 4 | 2 | 1 | 1 | 0 | 1 | 49 | 10 | 3 |
| 4 | 10 | 0 | 0 | 0 | 0 | 0 | 0 | 19 | 2 | 2 | 0 | 0 | 0 | 1 | 21 | 3 | 1 | 0 | 2 | 0 | 1 | 50 | 5 | 2 |
| 5 | 8 | 0 | 0 | 0 | 0 | 0 | 0 | 6 | 2 | 2 | 0 | 0 | 0 | 0 | 34 | 10 | 5 | 1 | 4 | 0 | 4 | 48 | 12 | 4 |
| 6 | 8 | 0 | 0 | 0 | 0 | 0 | 0 | 13 | 2 | 0 | 1 | 1 | 0 | 1 | 30 | 13 | 5 | 4 | 3 | 1 | 9 | 51 | 15 | 10 |
| 7 | 8 | 1 | 0 | 1 | 0 | 0 | 0 | 12 | 5 | 1 | 3 | 1 | 0 | 3 | 30 | 7 | 3 | 3 | 1 | 0 | 5 | 50 | 13 | 8 |
| 8 | 8 | 0 | 0 | 0 | 0 | 0 | 0 | 9 | 2 | 2 | 0 | 0 | 0 | 0 | 31 | 8 | 2 | 3 | 2 | 1 | 3 | 48 | 10 | 3 |
| 9 | 14 | 0 | 0 | 0 | 0 | 0 | 0 | 16 | 1 | 1 | 0 | 0 | 0 | 0 | 19 | 6 | 4 | 2 | 0 | 0 | 2 | 49 | 7 | 2 |
| 10 | 13 | 0 | 0 | 0 | 0 | 0 | 0 | 20 | 2 | 0 | 1 | 1 | 0 | 0 | 17 | 3 | 1 | 2 | 0 | 0 | 1 | 50 | 5 | 1 |
| 11 | 6 | 0 | 0 | 0 | 0 | 0 | 0 | 22 | 0 | 0 | 0 | 0 | 0 | 0 | 22 | 9 | 3 | 4 | 2 | 0 | 6 | 50 | 9 | 6 |
| 12 | 10 | 0 | 0 | 0 | 0 | 0 | 0 | 27 | 7 | 3 | 2 | 1 | 1 | 2 | 13 | 5 | 2 | 2 | 0 | 1 | 1 | 50 | 12 | 3 |
| 13 | 3 | 0 | 0 | 0 | 0 | 0 | 0 | 21 | 3 | 2 | 1 | 0 | 0 | 2 | 23 | 8 | 4 | 2 | 0 | 2 | 2 | 47 | 11 | 4 |
| 14 | 9 | 0 | 0 | 0 | 0 | 0 | 0 | 19 | 4 | 2 | 0 | 2 | 0 | 0 | 22 | 5 | 2 | 2 | 0 | 1 | 2 | 50 | 9 | 2 |
| 15 | 3 | 0 | 0 | 0 | 0 | 0 | 0 | 10 | 0 | 0 | 0 | 0 | 0 | 0 | 36 | 10 | 3 | 2 | 5 | 0 | 6 | 49 | 10 | 6 |
| 16 | 5 | 0 | 0 | 0 | 0 | 0 | 0 | 11 | 3 | 2 | 0 | 1 | 0 | 1 | 30 | 9 | 4 | 4 | 1 | 0 | 6 | 46 | 12 | 7 |
| Totals | 125 | 3 | 2 | 1 | 0 | 0 | 0 | 270 | 43 | 25 | 9 | 8 | 1 | 14 | 390 | 111 | 50 | 33 | 22 | 6 | 52 | 785 | 157 | 66 |
| % | | 2·4 | 1·6 | 0·8 | — | — | — | | 16 | 9·3 | 3·3 | 3 | 0·4 | 5·2 | | 28 | 13 | 8·5 | 5·6 | 1·5 | 13·3 | | 20 | 8·4 |

* Highest grade of tumours examined microscopically.

of various sites, and 32—miscellaneous benign nor malignant neoplasms. These 105 different tumours were distributed seemingly at random through the 16 groups. In so far as most of these tumours were incidental findings at necropsy, and not causes of death, and in so far as many of them were benign and/or slow growing, the incidence rates are not susceptible to actuarial analysis.

### DISCUSSION

In our study of the induction of malignant skin tumours by the regular application of carcinogens to mouse skin, we have found an approximately linear dose/response curve for NF, and a definitely non-linear dose/response curve for BP. We also found that combinations of NF and BP together gave a higher incidence rate than the sum of the incidence rates that they would have produced alone. Carcinogenesis is known to be a multi-stage process, and these findings suggest that NF acts mainly on only one rate-determining stage of the carcinogenic process whereas BP acts on more than one such stage.

The existence of a carcinogen-cocarcinogen interaction means that PAH in the inhuman environment at concentrations which are themselves subcarcinogenic might nevertheless be dangerous in the presence of other agents, particularly tobacco smoke.

For the purposes of analysing the results of the experiments reported, new statistical techniques based on actuarial analysis have been developed. The advantages—indeed the necessity—for using actuarial methods for analysing the results of survival experiments (*i.e.* most animals are still alive and well) is clearly exemplified in this paper. Our experience with these techniques also illustrates the limitation of actuarial analysis in that it cannot be applied to non-lethal slow-growing internal neoplasms, the presence of which is not reliably detectable until post mortem. Comparison of incidences of this class of neoplasm must be based on findings in killings of random samples of groups of animals.

We gratefully acknowledge the gift of Neutral Fraction of Cigarette Smoke Condensate by the Tobacco Research Council, and the generous financial support provided by the Medical Research Council and the Cancer Research Campaign in the form of Block Grants to this Institute. We also offer our thanks to many individuals who at one time or another helped with the experimental work, its interpretation and presentation. In this connection we mention especially Miss Janice Hotham, Miss Marjorie Butt, Mrs. Audrey Inglefield, Mr. Edward Sykes, and the staff of the histology department.

### REFERENCES

Bock, F. G., Moore, G. E. and Clark, P.C.—(1965*a*) *J. natn. Cancer Inst.*, **34**, 481.
Bock, F. G., Moore, G. E., Dowd, J. E. and Clark, P. C.—(1962) *J. Am. med. Ass.*, **181**, 668.
Bock, F. G., Schamberger, R. J. and Myers, H. K.—(1965*b*) *Nature, Lond.*, **208**, 584.
Day, T. D.—(1967) *Br. J. Cancer*, **21**, 56.
Dunn, T. B.—(1954) *J. natn. Cancer Inst.*, **14**, 1281.
Gellhorn, A.—(1958) *Cancer Res.*, **18**, 510.
Horton, A. W., Denman, D. T. and Trosset, R. P.—(1957) *Cancer Res.*, **17**, 758.
Kennaway, E. L. and Lindsey, A. J.—(1958) *Br. med. Bull.*, **14**, 124.
Peto, R. and Peto, J.—(1971) *Jl R. statist. Soc.*, in press.

PIKE, M. C. AND DOLL, R.—(1965) *Lancet*, i, 665.
PIKE, M. C. AND ROE, F. J. C.—(1963) *Br. J. Cancer*, **17**, 605.
POEL, W. E.—(1959) *J. natn. Cancer Inst.*, **22**, 19.
ROE, F. J. C.—(1962) *Nature, Lond.*, **194**, 1089.—(1965) *Fd Cosmet. Toxic.*, **3**, 707.
ROE, F. J. C., SALAMAN, M. H., COHEN, J. AND BURGAN, J. G.—(1959) *Br. J. Cancer*, **13**, 623.
SHUBIK, P. AND SAFFIOTTI, U.—(1962) *Natn. Cancer Inst. Monogr.*, **10**, 489.
WALTERS, M.A.—(1966) *Br. J. Cancer*, **20**, 148.
WYNDER, E. L., FRITZ, L. AND FURTH, N.—(1957) *J. natn. Cancer Inst.*, **19**, 361.
WYNDER, E. L. AND HOFFMANN, D.—(1961) *Cancer, N.Y.*, **14**, 1306.—(1963) *Dt. med. Wschr.*, **88**, 623.—(1967) ‘Tobacco and Tobacco Smoke: Studies in Experimental Carcinogenesis’. New York (Academic Press).